# A Testis-Specific Long Non-Coding RNA, *lncRNA-Tcam1*, Regulates Immune-Related Genes in Mouse Male Germ Cells

**DOI:** 10.3389/fendo.2017.00299

**Published:** 2017-11-02

**Authors:** Misuzu Kurihara, Kai Otsuka, Shin Matsubara, Akira Shiraishi, Honoo Satake, Atsushi P. Kimura

**Affiliations:** ^1^Graduate School of Life Science, Hokkaido University, Sapporo, Japan; ^2^Bioorganic Research Institute, Suntory Foundation for Life Sciences, Kyoto, Japan; ^3^Department of Biological Sciences, Faculty of Science, Hokkaido University, Sapporo, Japan

**Keywords:** long non-coding RNA, spermatogenesis, GC-2spd(ts) cells, gene activation, immune response

## Abstract

Spermatogenesis is precisely controlled by hormones from the hypothalamus–pituitary–gonadal axis and testis-specific genes, but the regulatory mechanism is not fully understood. Recently, a large number of long non-coding RNAs (lncRNAs) are found to be transcribed at each stage of meiosis of male germ cells, and their functions in spermatogenesis have yet to be fully investigated. *lncRNA-testicular cell adhesion molecule 1* (*lncRNA-Tcam1*) is a nuclear lncRNA which is specifically expressed in mouse male germ cells and presumed to play a role in gene regulation during meiosis. Here, we present the identification of potential target genes of *lncRNA-Tcam1* using spermatocyte-derived GC-2spd(ts) cells. Initially, 55 target gene candidates were detected by RNA-sequencing of two GC-2spd(ts) cell clones that were stably transfected with transgenes to express *lncRNA-Tcam1* at different levels. Expression of 21 genes of the candidates was found to be correlated with *lncRNA-Tcam1* at 7–14 postnatal days, when *lncRNA-Tcam1* expression was elevated. Subsequently, we examined expression levels of the 21 genes in other two GC-2spd(ts) clones, and 11 genes exhibited the correlation with *lncRNA-Tcam1*. Induction of *lncRNA-Tcam1* transcription using the Tet-off system verified that six genes, *Trim30a, Ifit3, Tgtp2, Ifi47, Oas1g*, and *Gbp3*, were upregulated in GC-2spd(ts) cells, indicating that *lncRNA-Tcam1* is responsible for the regulation of gene expression of the six genes. In addition, five of the six genes, namely, *Ifit3, Tgtp2, Ifi47, Oas1g*, and *Gbp3*, are immune response genes, and *Trim30a* is a negative regulator of immune response. Altogether, the present study suggests that *lncRNA-Tcam1* is responsible for gene regulation for the immune response during spermatogenesis.

## Introduction

Spermatogenesis is a process to generate spermatozoa, composed of meiosis and spermiogenesis (1, 2). In meiosis, some population of spermatogonia differentiate into spermatids, and the spermatids change their shapes to become spermatozoa through spermiogenesis. This process is controlled by the hypothalamus–pituitary–gonadal axis. The pituitary secretes gonadotropin in response to gonadotropin releasing hormone from hypothalamus, by which steroid production is stimulated in testis, leading to normal progression of spermatogenesis (3–5). In addition, many testis-specific genes are activated in germ cells during meiosis and play important roles in spermatogenesis (6–14). However, the regulatory mechanism of spermatogenesis is not fully understood. To date, various long non-coding RNAs (lncRNAs) have been identified in the testis (15, 16), and some of them were shown to be upregulated by sex steroids (17–21). These findings suggest that lncRNAs are involved in the regulation of spermatogenesis. Nevertheless, the physiological significance of lncRNAs and the molecular mechanisms of their actions in the testis are largely unknown.

Long non-coding RNAs are molecules that are longer than 200 nucleotides and function without being translated (22). They are localized in nuclei, cytosols, and extracellular vesicles (23–25), and involved in various biological events such as development (26, 27), cell differentiation (28, 29), immune response (30, 31), and cell metabolism (32, 33). lncRNAs exert their effects *via* controlling gene expression at various levels, i.e., transcription, mRNA stability, and translation (22, 26, 34). In nuclei, lncRNAs are frequently involved in the transcriptional control of protein-coding genes (35). Thus, lncRNAs are presumed to be essential for transcriptional regulation of meiotic genes during spermatogenesis, while biological functions of testicular lncRNAs have not been well investigated.

We have studied the mechanism of gene activation during meiosis by using the mouse *testicular cell adhesion molecule 1* (*Tcam1*) locus as a model. The *Tcam1* gene is specifically expressed in spermatocytes and encodes a cell adhesion molecule, which is dispensable for sperm generation (36, 37). We previously identified a dual promoter–enhancer, which enhanced the *Tcam1* gene transcription and drove the *SWI/SNF-related, matrix-associated, actin-dependent regulator of chromatin, subfamily d, member 2* (*Smarcd2*) gene and *lncRNA-Tcam1* (38). *lncRNA-Tcam1* was a novel testis-specific lncRNA which was 2.4-kb nucleotide long and localized to nuclei of germ cells but not to Leydig and Sertoli cells (38). Such germ cell-specific localization suggests that *lncRNA-Tcam1* plays a role in specific gene activation during meiosis.

To study molecular mechanisms in various types of cells, the *in vitro* cell culture system is useful, and some cell lines were established from male germ cells (39). The GC-2spd(ts) cell line was established from mouse spermatocytes using the SV40 large T antigen and the temperature-sensitive P53 mutant gene (40), and was widely used due to their ability of differentiation. After multiple cell passages, the cells still maintain some properties as male germ cells and are widely used for examining mechanisms in germ cells (41–48). Thus, the GC-2spd(ts) cell line is one of the most useful models for the study of the regulatory mechanism in male germ cells.

In this paper, we show that *lncRNA-Tcam1* induces immune-related genes in GC-2spd(ts) cells, which shed new light on novel regulatory roles of lncRNAs in the testis.

## Materials and Methods

### Animals

The mice (C57/BL6) were maintained at 25°C with a photoperiod of 14:10 h light:dark with free access to food and water. Testes were isolated from 7- to 56-day-old mice and stored at −80°C until use. Experimental procedures used in this study were approved by the Institutional Animal Use and Care Committee at Hokkaido University.

### Cell Culture

**Figure 1:**
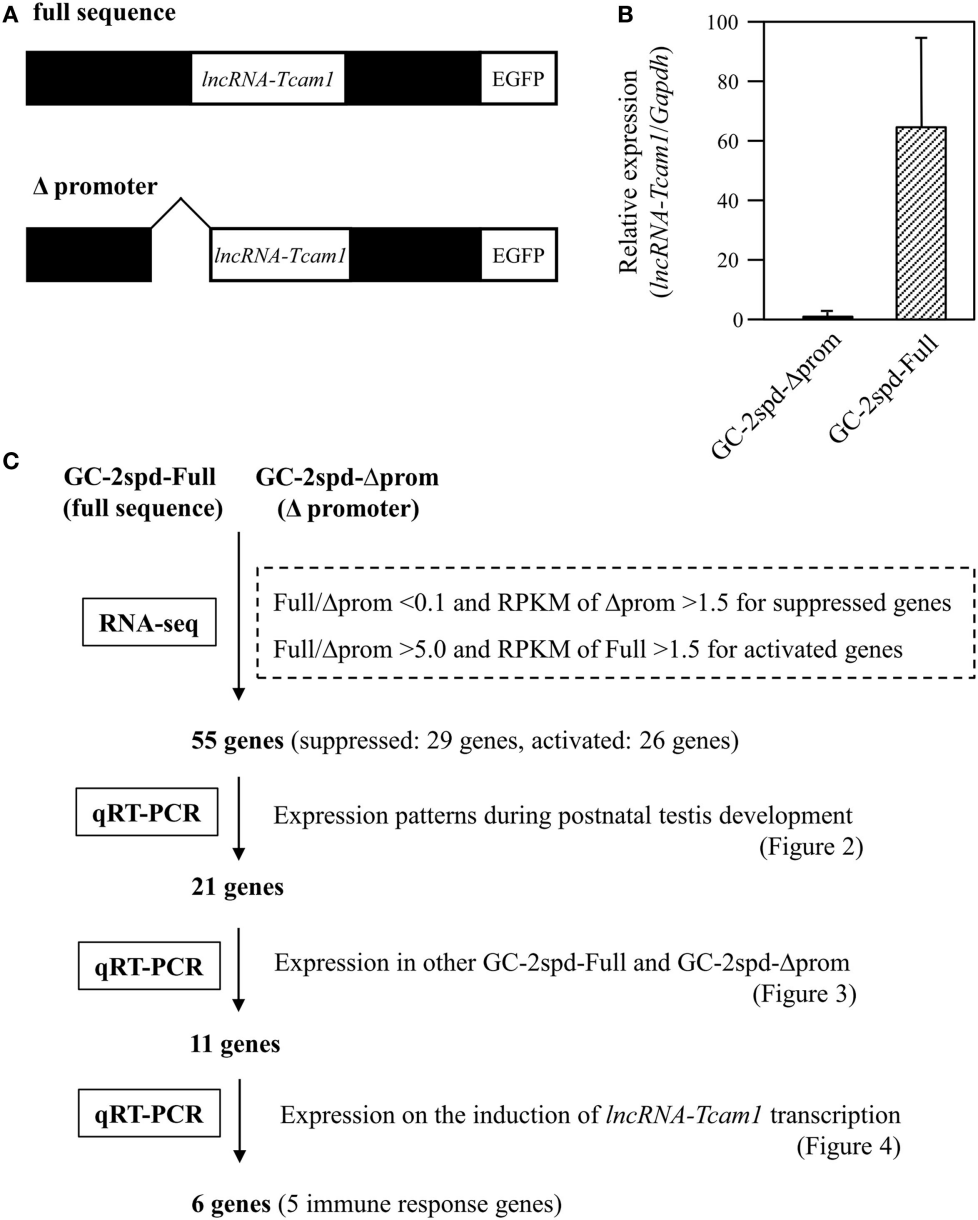
A strategy to identify potential target genes of *long non-coding RNA-testicular cell adhesion molecule 1* (*lncRNA-Tcam1*). **(A)** Constructs used for establishing GC-2spd(ts) cell clones that expressed *lncRNA-Tcam1* at different levels. A 6.9-kb sequence encompassing the upstream and downstream regions of *lncRNA-Tcam1* was obtained from a mouse BAC clone, and connected to the enhanced green fluorescent protein (EGFP) gene (full sequence). A 0.8-kb sequence corresponding to the *lncRNA-Tcam1* promoter was deleted by restriction digestion (Δpromoter). The reporter EGFP gene was used to investigate the transcriptional regulation activity of the lncRNA promoter sequence in our previous study. Each construct was transfected into GC-2spd(ts) cells, and stable cell clones were established by the limited dilution method. **(B)** Expression of *lncRNA-Tcam1* in GC-2spd-Full and GC-2spd-Δprom. GC-2spd-Δprom was derived from the cells transfected with “Δpromoter” and used as a clone expressing *lncRNA-Tcam1* at a low level. GC-2spd-Full was a stably transfected clone with “full sequence” and used as the cells expressing *lncRNA-Tcam1* at a high level. Total RNA was isolated from each clone, and the expression level of *lncRNA-Tcam1* was measured by quantitative reverse transcription-polymerase chain reaction (qRT-PCR). The value was normalized to the *Gapdh* mRNA level, and the level in GC-2spd-Δprom was set to 1.0. The data are presented as mean value ± SD from three independent experiments. **(C)** An outline of the screening process to identify potential target genes of *lncRNA-Tcam1* in this study.

GC-2spd(ts) and its derivatives were cultured in Dulbecco Modified Eagle medium containing 10% fetal bovine serum, 100 U/ml penicillin, 100 μg/ml streptomycin, and 292 μg/ml l-glutamine (Invitrogen, Carlsbad, CA, USA) (38, 40). We used two types of GC-2spd(ts)-derived cell clones: lncRNA-6.9kb-EGFP clones that contained the intact sequence of the *lncRNA-Tcam1* locus (full sequence) and ΔCNS1-lncRNA-EGFP clones that lacked the *lncRNA-Tcam1* promoter (Δpromoter) (Figure 1A).

### RNA Preparation and Quantitative Reverse Transcription-Polymerase Chain Reaction (qRT-PCR)

Isolation of total RNAs, treatment with DNase, and qRT-PCR were done as previously described (38, 49). To detect *lncRNA-Tcam1*, we used KOD SYBR qPCR Mix (Toyobo, Osaka, Japan), and the other transcripts were measured with Power SYBR Green Master Mix (Applied Biosystems, Foster City, CA, USA). Primer pairs are listed in Table 1.

**Table 1:** Oligonucleotide primers used in this study.

Designation	Forward	Reverse
lncRNA-Tcam1	5′-GACTGTCTGGGCAGAGTGAA-3′	5′-GAACCCAAGCAAAGCTGTAAAC-3′
Aip	5′-GAGGACGGGATCCAAAAGC-3′	5′-CTGTGCAGCGTCCGAAAGT-3′
Gapdh	5′-TGCACCACCAACTGCTTAGC-3′	5′-GGCATGGACTGTGGTCATGAG-3′
H19	5′-TGGGAAAAGTGAAAGAACAG-3′	5′-GTGTGATGGAGAGGACAGAA-3′
Ren1	5′-ATCCTTTATCTCGGCTCCTA-3′	5′-ACCTGGCTACAGTTCACAAC-3′
Igfbp5	5′-CTGACCCTCTACCTTCCTTT-3′	5′-TGAGCAGACTTTCTTGGTTT-3′
Gpx7	5′-GTTCACCACCAGGGAAAC-3′	5′-GCAGGACTTCTACGACTTCA-3′
Bmyc	5′-GACCACGACGGTGATAGCTT-3′	5′-TCCAGCTTGGAGACCAGCTT-3′
Avpr1a	5′-AAGATCCGCACAGTGAAGAT-3′	5′-GTTCAAGGAAGCCAGTAACG-3′
Iigp1	5′-AAGAGCACACCGAGGGCTAT-3′	5′-GCTGGAGGGCAAATCATTAT-3′
Oas2	5′-CTACTGACCCAGATCCAGAA-3′	5′-TGGCACTTTCCAAGGCTGTA-3′
Trim30a	5′-GGACAGGTTACTTCCTCCTT-3′	5′-GTCTCTTGGTTGGTATCTGA-3′
Ifit3	5′-CCAGCAGCACAGAAACAGAT-3′	5′-GACATACTTCCTTCCCTGAA-3′
Hoxd13	5′-GGAACAGCCAGGTGTACTGT-3′	5′-TCATTCTCCAGTTCTTTGAG-3′
Tgtp2	5′-ATGGCTCTGTATGGTAGAAG-3′	5′-CAGAACTCCACACCTCATGT-3′
Mx2	5′-TTCAAGGAACACCCTCATT-3′	5′-CTCTGCGGTCAGTCTCTCT-3′
Fabp5	5′-ACGGGAAGGAGAGCACGATA-3′	5′-GCAGGTGGCATTGTTCAT-3′
Ifi47	5′-GTGAGAAACAGACCCGGTAT-3′	5′-ATGCCTCCTGCCTTACTGAT-3′
Enpp5	5′-CCTTGTTTCTGCCTCCTCTT-3′	5′-AGCCGAATGGCATAGAGTAG-3′
Oas1g	5′-CCAGATGAGGATGGTGTAGA-3′	5′-TCAGGAGGTGGAGTTTGAT-3′
Usp18	5′-CTCGGTGATACCAAGGAACA-3′	5′-ACCAAAGTCAGCCATCCCAA-3′
Gbp3	5′-GGATTCTTGAGCAGATAGCA-3′	5′-ATACCCTTGGTTTCGGATT-3′
Ifi44	5′-GGTTTGATGTGATTGGTTTC-3′	5′-CTGCCATTTATTCTGTGTGA-3′
Ifit1	5′-GAGTTCTGCTCTGCTGAAAA-3′	5′-AGGAACTGGACCTGCTCTGA-3′

### RNA-Sequencing (RNA-seq) Analysis

Total RNAs were isolated from a lncRNA-6.9kb-EGFP clone (full sequence) and a ΔCNS1-lncRNA-EGFP clone (Δpromoter) using ISOGEN II (Nippongene, Tokyo, Japan) according to the manufacturer’s instruction. The RNAs were further purified by phenol/chloroform–isoamylalcohol extraction and ethanol precipitation, and treated with TurboDNase (Applied Biosystems). The quality of the RNA samples was evaluated using BioAnalyzer (Agilent Technologies, Palo Alto, CA, USA) with RNA6000 Nano Chip. A 500-ng aliquot of total RNA from each sample was used to construct cDNA libraries using TruSeq Stranded mRNA Sample preparation kit (Illumina, San Diego, CA, USA), according to the manufacturer’s instructions. The resulting cDNA library was validated using BioAnalyzer with DNA1000 Chip and quantified using Cycleave PCR Quantification Kit (Takara Bio Inc., Otsu, Japan). 101-cycle of single-end sequencing was performed using HiSeq1500 (Illumina) in the rapid mode. Total reads were extracted with CASAVA v1.8.2 (Illumina). Then, PCR duplicates, adaptor sequences and low quality reads were removed from the extracted reads as follows. Briefly, if the first 10 bases of the two reads were identical and the entire reads exhibited >90% similarity, the reads were considered PCR duplicates. Remaining reads were then aligned with Bowtie version 2.2.3 to the mouse genome, mm10, which was downloaded from UCSC (https://genome.ucsc.edu/). The expression level of each gene was calculated as gene-specific reads per million total reads (RPM).

### Plasmid Construction

A PiggyBac-based plasmid for induction of *lncRNA-Tcam1* transcription was generated as follows. A full length of *lncRNA-Tcam1* was amplified by PCR with mouse genome DNA and KOD FX Neo (Toyobo) using a primer pair, 5′-CCGGAGGAGCGGGAGCGGAA-3′ and 5′-CACCGGAAAACAGCTTTAAT-3′. We used the genome DNA as a template because *lncRNA-Tcam1* contained no intron. The product was subcloned into a pBluescript II vector (Stratagene, La Jolla, CA, USA) at *Eco*RV site, and the resulting plasmid was checked by DNA sequencing method. This plasmid was digested with *Sal*I, blunted by T4 DNA polymerase (Takara), and further digested with *Not*I. The *lncRNA-Tcam1* fragment was purified and inserted into pPBhCMV*1-cHA-pPA at the *Not*I/blunted *Xho*I site. The completed plasmid was checked by DNA sequencing and named pPBhCMV*1-lncTcam1-pPA. All plasmids for establishing the Tet-off system were kindly gifted by Dr. Kazuhiro Murakami (50).

### Establishment of the Tet-Off System and Induction of *lncRNA-Tcam1* Transcription

GC-2spd(ts) cells were co-transfected with pPBhCMV*1-lncTcam1-pPA, pPBCAGtTA-IN, pPyCAG-PBase, and pKO-Select-Puro plasmids using GeneJuice transfection reagent (Merck, Darmstadt, Germany) and selected with 3 μg/ml puromycin for 14 days. The selected cells were kept in the presence of 1 μg/ml doxycycline (Dox). For inducing *lncRNA-Tcam1* transcription, the cells were spread onto 35-mm dishes, and Dox was removed on the next day.

### Statistical Analysis

**Figure 2:**
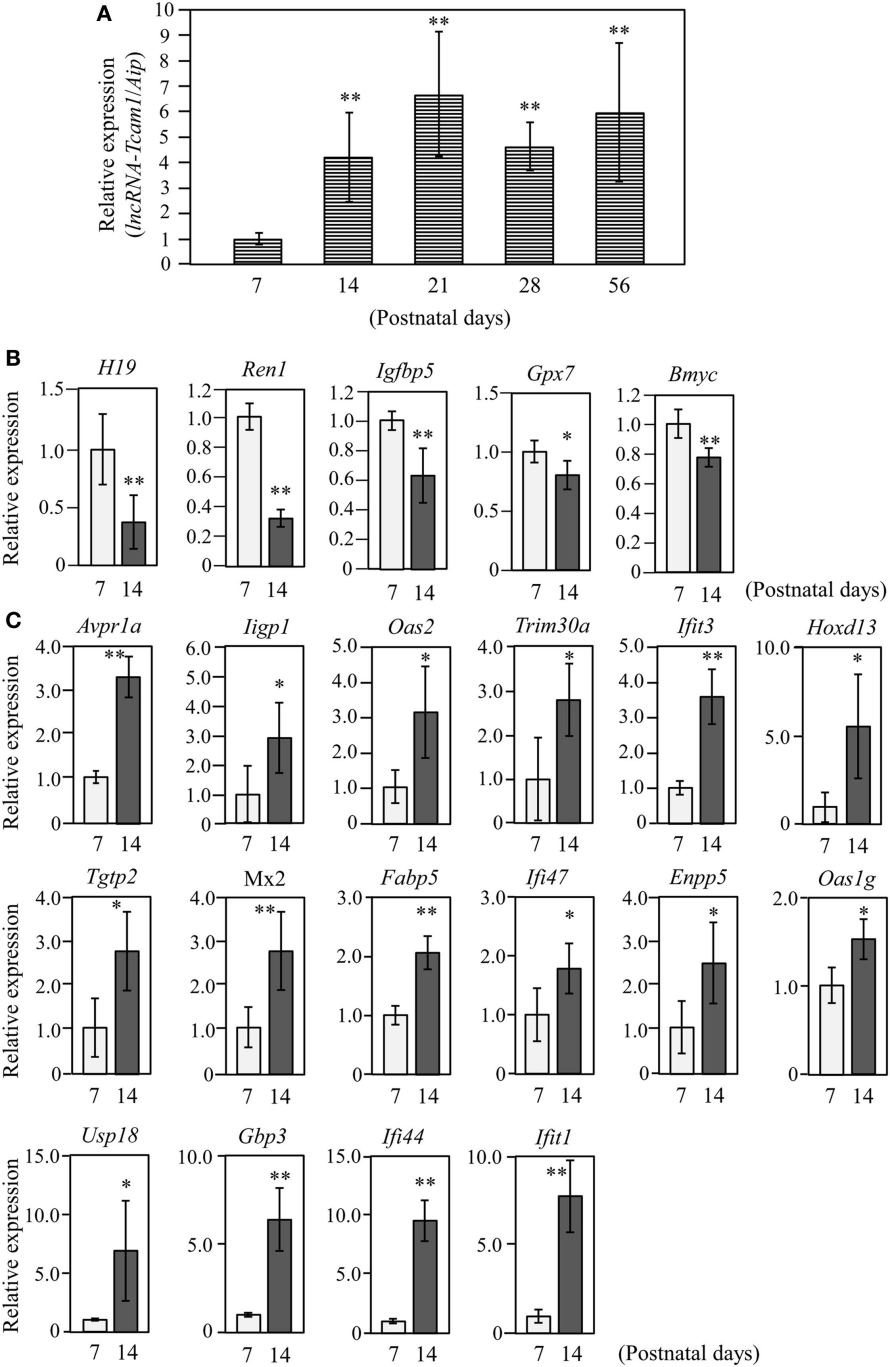
Expression of *long non-coding RNA-testicular cell adhesion molecule 1* (*lncRNA-Tcam1*) and its target gene candidates in postnatal testes. **(A)** *lncRNA-Tcam1* expression in mouse testes at various postnatal stages. Total RNAs were purified from whole testes of mice at indicated ages. Expression levels of *lncRNA-Tcam1* were measured by quantitative reverse transcription-polymerase chain reaction (qRT-PCR) as in Figure 1B, and the data were normalized to the housekeeping *Aip* gene. The value at day 7 was set to 1.0. **(B)** Expression of five candidate genes that were presumed to be suppressed by *lncRNA-Tcam1* in testes of 7- and 14-day-old mice. qRT-PCR was performed, and the data were normalized to *Aip*. The level at day 7 was set to 1.0. Light gray bars represent expression levels at 7 days of age, and light black bars indicate those at 14 days. **(C)** Expression of 16 candidate genes that were presumed to be activated by *lncRNA-Tcam1*. qRT-PCR was performed and the data were normalized as in **(B)**. All experiments were performed four times with two different RNA sets from testes, and the averages ± SD are indicated. The statistical significance was analyzed by one-way analysis of variance followed by Dunnett’s test in **(A)** and by Student’s *t*-test in **(B,C)**. *P < 0.05, **P < 0.01 compared to day 7.

**Figure 3:**
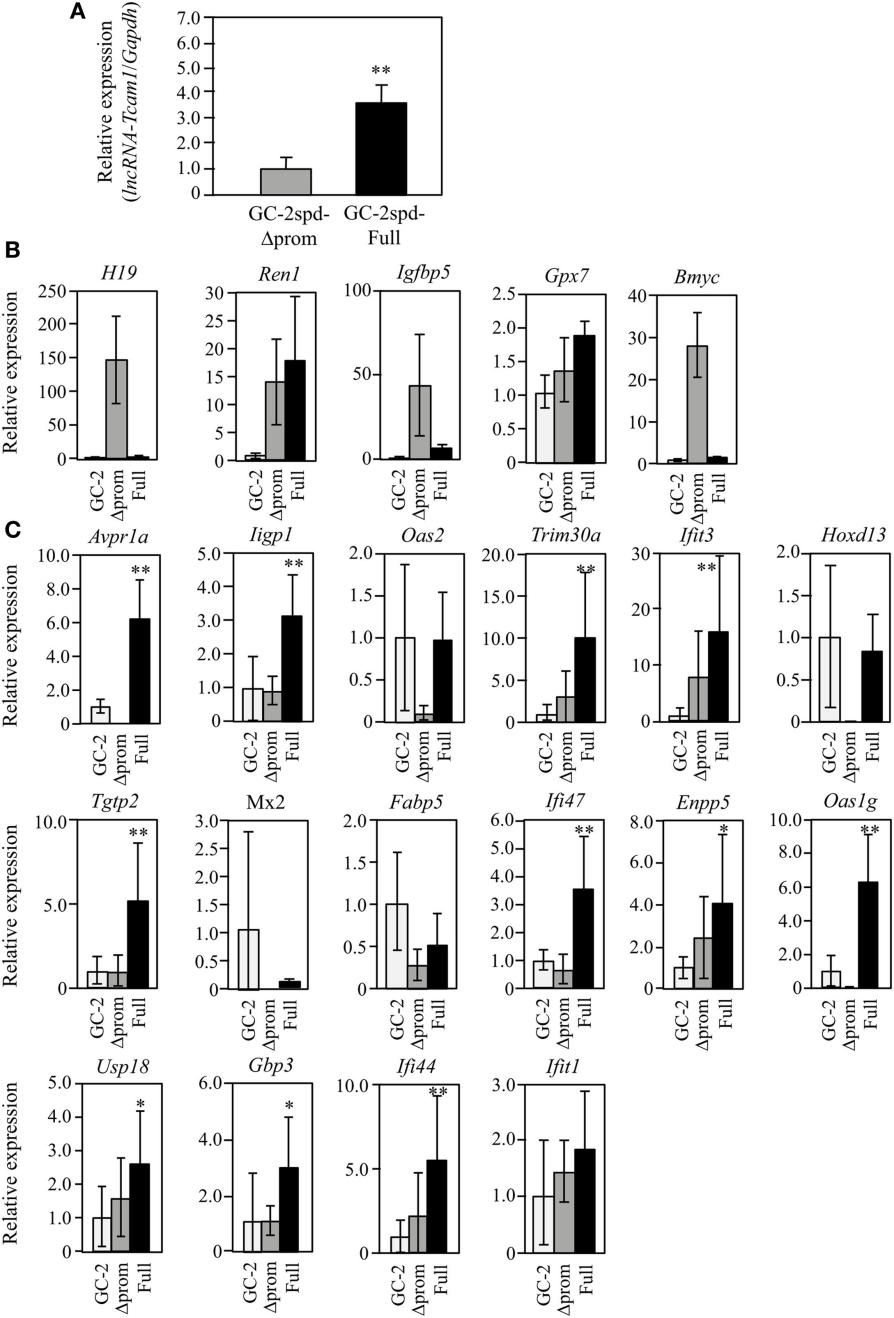
Expression of *lncRNA-testicular cell adhesion molecule 1* (*lncRNA-Tcam1*) and its target gene candidates in GC-2spd-Full and GC-2spd-Δprom different from those used for RNA-sequencing. **(A)** *lncRNA-Tcam1* expression in GC-2spd-Full and GC-2spd-Δprom. The expression level of *lncRNA-Tcam1* was measured by quantitative reverse transcription-polymerase chain reaction (qRT-PCR), and the data were normalized to *Gapdh*. The level in GC-2spd-Δprom was set to 1.0. The data are the means ± SD from three independent experiments, and the statistical significance was evaluated by Student’s *t*-test. **P < 0.01 compared to GC-2spd-Δprom. **(B)** Expression of five candidate genes that were presumed to be suppressed by *lncRNA-Tcam1* in GC-2spd(ts) cells without being transfected, GC-2spd-Δprom, and GC-2spd-Full. qRT-PCR was performed, and the data were normalized to *Gapdh*. The level in GC-2spd(ts) cells was set to 1.0. White bars represent the data from GC-2spd(ts) cells without transfection, and gray and black bars show the values from GC-2spd-Δprom and GC-2spd-Full, respectively. **(C)** Expression of 16 candidate genes that were presumed to be activated by *lncRNA-Tcam1*. qRT-PCR was performed, and the data were normalized to *Gapdh*. The data are presented as in **(B)**. The data for GC-2spd(ts) cells are the averages ± SD from four independent experiments, and the other data represent the means ± SD from three independent experiments. The statistical significance was evaluated by one-way analysis of variance followed by Dunnett’s test in **(B,C)**. *P < 0.05, **P < 0.01 compared to GC-2spd(ts) cells without transfection.

**Figure 4:**
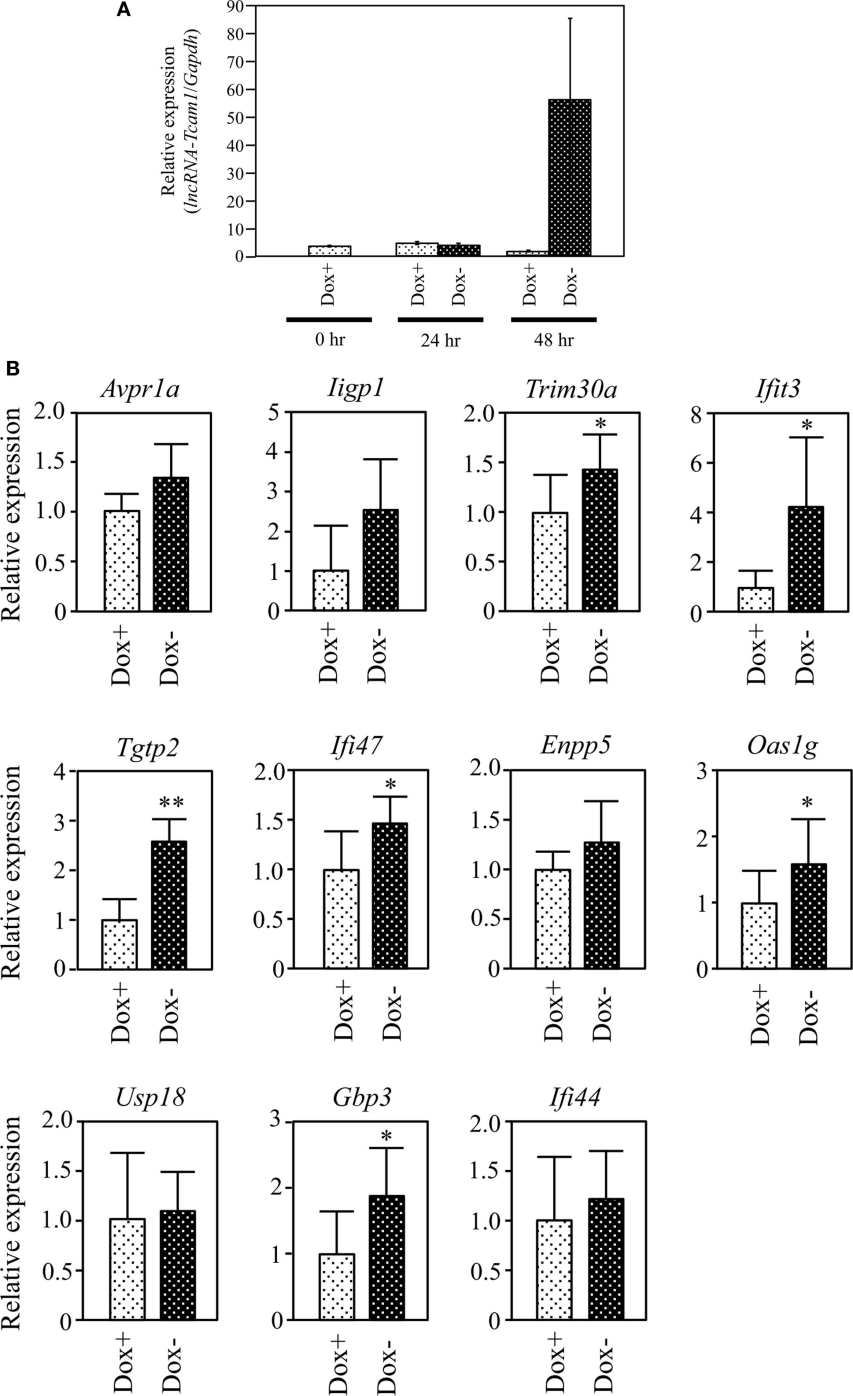
Expression of target gene candidates in response to induction of *long non-coding RNA-Tcam1* (*lncRNA-Tcam1*) transcription by the Tet-off system. **(A)** Induction of *lncRNA-Tcam1* transcription by the Tet-off system. GC-2spd(ts) cells were transfected with the construct for *lncRNA-Tcam1* overexpression in response to the doxycycline (Dox) removal. After the selection of successfully transfected cells with puromycin, they were cultured in the presence (Dox+) or absence of Dox (Dox−) for 24 or 48 h. The cells were collected, and quantitative reverse transcription-polymerase chain reaction (qRT-PCR) was performed to measure the *lncRNA-Tcam1* level using *Gapdh* as an internal control. The data represent the means ± SD from three independent experiments. A dramatic increase of *lncRNA-Tcam1* transcription was observed in the Dox− sample at 48 h after the induction. **(B)** Expression of candidate genes at 48 h after the induction. qRT-PCR was performed, and the data were normalized to *Gapdh*. The values from Dox− samples were further normalized to those from Dox+ samples and expressed as fold-increases. The data represents the means ± SD from five independent experiments. The statistical significance was analyzed by Student’s *t*-test. *P < 0.05, **P < 0.01 compared to Dox+ samples.

The results were expressed as means ± SD. The data were analyzed by one-way analysis of variance (ANOVA) followed by Dunnett’s test (Figures 2A, and 3B,C) or by Student’s *t*-test (Figures 2B,C, 3A, and 4B) using Microsoft Excel statistical analysis functions (Microsoft Corporation, Redmond, WA, USA).

## Results

### RNA-seq with GC-2spd(ts) Cell Clones Expressing *lncRNA-Tcam1* at Different Levels

To identify potential target genes that were regulated by *lncRNA-Tcam1*, we used GC-2spd(ts) cell clones we established in a previous study (38). In these lines, we stably transfected a construct, which contained a 6.9-kb sequence encompassing the *Tcam1* promoter and the *lncRNA-Tcam1* region (full sequence) or the same sequence without the *lncRNA-Tcam1* promoter (Δpromoter), connected to the enhanced green fluorescent protein (EGFP) gene (Figure 1A). *lncRNA-Tcam1* was expressed at higher levels in the clones with “full sequence” than those with “Δpromoter” (38), and hereafter, we designated “GC-2spd-Full” and “GC-2spd-Δprom,” respectively. We chose a representative cell clone from each cell line, and the GC-2spd-Full and GC-2spd-Δprom clones showed 63-fold difference in expression levels of *lncRNA-Tcam1* by qRT-PCR (Figure 1B). We expected that target genes of this lncRNA should be expressed at different levels between them.

To determine the genes that were differentially expressed between GC-2spd-Full and GC-2spd-Δprom, we performed the RNA-seq analysis. 101-cycle of single-end sequencing using HiSeq 1500 yielded 13,054,029 and 13,194,037 reads for GC-2spd-Full and GC-2spd-Δprom, respectively, and 92.25 and 90.23% each of which was mapped to the mouse genome (mm10). The resultant fastq files were deposited on NCBI SRA database (accession no. SRR5435658 and SRR5435659). Subsequently, we compared the gene expression levels between GC-2spd-Full and GC-2spd-Δprom, and picked protein-coding genes that were expressed at lower levels than 0.1-fold in GC-2spd-Full as candidate genes negatively regulated by *lncRNA-Tcam1*, and those expressed at higher levels than fivefold in GC-2spd-Full as positively regulated genes. We then selected the gene whose RPKM value was higher than 1.5 in GC-2spd-Δprom or GC-2spd-Full. As a result, 29 genes were chosen as candidates potentially suppressed by *lncRNA-Tcam1*, and 27 genes as those activated by the lncRNA. However, after this selection, we found that one potentially activated gene was not detected in GC-2spd-Full by qRT-PCR, and eliminated it from candidate genes. Consequently, a total of 55 genes (29 potentially suppressed and 26 activated genes, Tables S1 and S2 in Supplementary Material) were subject to further analysis. An outline of the screening of candidate genes is shown in Figure 1C.

### Expression of Candidate Genes in Postnatal Testes

The target genes should be expressed correlatively to *lncRNA-Tcam1* in the testis. We thus examined the expression of these potential target genes during postnatal testis development. We collected testes at 7–56 days after birth and measured the *lncRNA-Tcam1* level by qRT-PCR. The transcript of *lncRNA-Tcam1* was detected in all samples, and a significant increase was observed between day 7 and day 14 after birth (Figure 2A). The absence of *lncRNA-Tcam1* expression in somatic cells in the mouse testis (38) led to the presumption that the transcription of *lncRNA-Tcam1* is initiated in spermatogonia and increased in primary spermatocytes. Then, we examined expression levels of the 55 candidate genes (29 potentially downregulated and 26 upregulated) in the testis at 7 days and 14 days after birth by qRT-PCR, to compare their levels in spermatogonia and primary spermatocytes. Among the genes that might be suppressed by *lncRNA-Tcam1*, 5 genes showed significant decreases during this period (Figure 2B), and 16 genes out of potentially activated candidates exhibited significant increases (Figure 2C). The expression patterns of genes that were not correlated with *lncRNA-Tcam1* is presented in Figures S1 and S2 in Supplementary Material. Signals for four downregulated candidates, *Cyclin D2* (*Ccnd2*), *CD55 molecule, decay accelerating factor for complement* (*Cd55*), *Syndecan 3* (*Sdc3*), and uc009oek.1, and an upregulated candidate, *2′-5*′ *oligoadenylate synthetase 3* (*Oas3*), were not detected by this analysis. From these data, 21 genes (5 genes down and 16 genes up) correlated with *in vivo lncRNA-Tcam1* expression were forwarded to the next analysis as target gene candidates.

### Expression of Candidate Genes in GC-2spd(ts) Cell Clones Different from Those Used for RNA-seq

To further validate the RNA-seq and postnatal testis data, the expression was also examined using GC-2spd(ts) clones that were different from those used for RNA-seq. This would help us to select the real targets, because the properties might be different between cell clones as a result of the process to establish stable cells and pick the clones. qRT-PCR demonstrated that *lncRNA-Tcam1* was expressed at a 3.5-fold higher level in GC-2spd-Full than in GC-2spd-Δprom (Figure 3A). We assessed the expression of 21 candidate genes using these two clones and GC-2spd(ts) cells without being transfected. By comparing to cells without being transfected, false-positive genes (due to the establishment of cell clones) could be excluded. Among five genes potentially suppressed by *lncRNA-Tcam1, H19, imprinted maternally expressed transcript* (*H19*), *Insulin-like growth factor binding protein 5* (*Igfbp5*), and *Brain expressed myelocytomatosis oncogene* (*Bmyc*) were expressed at much higher levels in GC-2spd-Δprom than in GC-2spd-Full, but they were expressed at very low levels in GC-2spd(ts) cells without transfection (Figure 3B), indicating that these three genes were artifacts. Expression of *Renin 1 structural* (*Ren1*) and *Glutathione peroxidase 7* (*Gpx7*) genes was positively correlated with *lncRNA-Tcam1* (Figure 3B), which was inconsistent with the genes suppressed by *lncRNA-Tcam1*. Therefore, the five genes were unlikely to be targets of *lncRNA-Tcam1*.

Among 16 genes, expression levels of five genes [*2′-5*′ *oligoadenylate synthetase 2* (*Oas2*), *Homeobox D13* (*Hoxd13*), *MX dynamin-like GTPase 2* (*Mx2*), *Fatty acid binding protein 5, epidermal* (*Fabp5*), and *Interferon-induced protein wit tetratricopeptide repeats 1* (*Ifit1*)] were not significantly higher in GC-2spd-Full than in GC-2spd(ts) cells (Figure 3C), excluding the possibility that these genes were targets of *lncRNA-Tcam1*. On the other hand, 11 genes were expressed at significantly higher levels in GC-2spd-Full than in GC-2spd(ts) cells, and their expression was higher in GC-2spd-Full than in GC-2spd-Δprom (Figure 3C). Collectively, these expression profiles detected 11 potential genes upregulated by *lncRNA-Tcam1* and they were subjected to further analysis.

### Identification of Genes Upregulated by *lncRNA-Tcam1*

We finally examined whether *lncRNA-Tcam1* could activate the 11 candidate genes in the Tet-off system. We constructed the plasmid in which the hCMV*1 promoter induced *lncRNA-Tcam1* transcription in response to the Dox removal, and stably transfected GC-2spd(ts) cells with it. qRT-PCR of the stable transfectants confirmed that the expression of *lncRNA-Tcam1* was 57-fold induced exclusively at 48 h after removal of Dox (Figure 4A).

We examined expression levels of the 11 candidate genes 48 h after the Dox removal by qRT-PCR. We set the value from the control cells that were cultured for 48 h with Dox to 1.0 and calculated the levels of candidates. six genes, *Tripartite motif-containing 30A* (*Trim30a*), *Interferon-induced protein wit tetratricopeptide repeats 3* (*Ifit3*), *T cell-specific GTPase 2* (*Tgtp2*), *Interferon gamma inducible protein 47* (*Ifi47*), *2′-5*′ *oligoadenylate synthetase 1G* (*Oas1g*), and *Guanylate binding protein 3* (*Gbp3*), exhibited significantly higher expression in the cells without Dox, and their fold-increases were 1.46, 3.37, 2.43, 1.44, 1.49, and 1.69, respectively (Figure 4B). We confirmed significant levels of expression of the six genes in the germ cell fraction from adult mouse testes (Table S3 in Supplementary Material). Interestingly, five genes of the final six candidates, namely, *Ifit3, Tgtp2, Ifi47, Oas1g*, and *Gbp3*, were immune response genes, and *Trim30a* was a negative-feedback regulator of immune response (Table 2). Collectively, these data indicated that *lncRNA-Tcam1* is responsible for the regulation of some immune response pathways during spermatogenesis.

**Table 2:** Genes potentially activated by *lncRNA-Tcam1*.

Gene	UCSC-ID	Description	Relation to immune response	Chromosome
Trim30a	uc009iwh.1	Tripartite motif-containing 30A	Negative-feedback regulator	7
Ifit3	uc008hgo.1	Interferon-induced protein with tetratricopeptide repeats 3	Immune response gene	19
Tgtp2	uc007ipp.2	T cell-specific GTPase 2	Immune response gene	11
Ifi47	uc007ipq.1	Interferon gamma inducible protein 47	Immune response gene	11
Oas1g	uc008zii.1	2′-5′ oligoadenylate synthetase 1G	Immune response gene	5
Gbp3	uc008rov.1	Guanylate binding protein 3	Immune response gene	3

## Discussion

Among various mouse and human tissues, the testis contains large numbers of lncRNAs (15, 16), as evidenced by recent transcriptomic analyses identifying many lncRNAs in different postnatal testes or fractionated germ cells (51–53). However, their functions in spermatogenesis are largely unknown in mammals, and we revealed the upregulation of gene expression of immune-related genes by mouse male germ cell-specific *lncRNA-Tcam1* using spermatocyte-derived GC-2spd(ts) cells as a model.

We performed RNA-seq of GC-2spd(ts) cell clones that expressed *lncRNA-Tcam1* at different levels and selected 55 genes as initial candidates of its targets. Then, we investigated (1) their expression in testes at different developmental stages, (2) their expression in GC-2spd(ts) clones different from those used for RNA-seq, and (3) the response to induction of *lncRNA-Tcam1* transcription by the Tet-off system. Eventually, these analyses detected six immune-related genes, *Trim30a, Ifit3, Tgtp2, Ifi47, Oas1g*, and *Gbp3* that were upregulated by *lncRNA-Tcam1*. Taken together, these results suggest that *lncRNA-Tcam1* participates in the functional regulation of the mouse testis *via* upregulation of the six genes during spermatogenesis.

In our previous study, we reported that *lncRNA-Tcam1* expression was unlikely to be related to the regulation of neighboring genes (38), and our present data support this. Four target genes, *Trim30a, Ifit3, Oas1g*, and *Gbp3*, were located on different chromosomes from *lncRNA-Tcam1*, and the other two genes, *Tgtp2* and *Ifi47*, were at the same locus on chromosome 11 but approximately 57 Mb distant from the lncRNA (Table 2). Therefore, *lncRNA-Tcam1* does not control its neighboring genes. How this lncRNA activates the six genes is not clear, but the *lncRNA-Tcam1* locus may spatially locate at positions close to its target gene loci in nuclei of germ cells or the *lncRNA-Tcam1* transcript may bind to its target loci in response to immune signals. Additionally, the modes of action of *lncRNA-Tcam1* in recognition of and binding to targets may be similar to those of other lncRNAs related to immune response (54, 55). Such studies are currently in progress.

Of particular interest is that the five target genes were immune response genes. *Ifit3* and *Oas1g* have antiviral functions in some tissues including the lung and liver (56, 57), and *Tgtp2, Ifi47*, and *Gbp3* contributes to defense against bacteria and protozoa (58–60). Interestingly, *Ifi47* and *Gbp3* were reported to play some roles in defense against *Toxoplasma Gondii* (58, 60), which could impair spermatogenesis by infecting the rat testis (61). In addition, *Trim30a* is a negative-feedback regulator of immune response against virus (62). Although their functional roles in the testis remain unknown (58, 60, 63), these findings suggest that *lncRNA-Tcam1* participates in immune response to multiple pathogenic microbes by activating these genes during meiosis, and thereby contributes to normal progression of spermatogenesis regulated by the endocrine system.

The physiological significance of some of the target genes in immune defense has been demonstrated by several studies. Interleukin 17a was elevated by infection of rhinovirus in the lung, and led to upregulation of the *Oas1g* gene, which caused the degradation of viral RNAs (64). Dengue virus infection in lung epithelial cells upregulated the *Ifit3* gene, which suppressed the virus production (65). In dendritic cells, infection of *Listeria monocytogenes* increased expression of *Tgtp2* and *Oas1g* genes to eventually reduce the bacterial titer (66). All of these indicate that the genes are responsive to various types of infection and contribute to immune defense *in vivo*, which suggests that they also play important roles in the immune response in the testis.

The testis is an immunoprivileged tissue and has evolved the innate immune system (67, 68). The innate immunity involves the interferon response, and key molecules for that are Toll-like receptors (TLRs). Activation of TLRs induces the interferon production and increases expression of interferon-responsive genes (67). Because mouse spermatogonia and primary spermatocytes constitutively express one of the TLR genes, *Tlr3*, and interferon receptor genes (69, 70), TLR3-mediated immune pathway is likely to be active in the testis. Notably, the five immune responsive genes are known to be responsive to interferon, so they may be activated through the TLR3 pathway in germ cells when the testis is infected by some pathogens. Therefore, *lncRNA-Tcam1* may regulate some innate immune responses by controlling the interferon-responsive genes during meiosis.

It is now well known that a wide variety of lncRNAs are expressed in the testis (15, 16, 71), but their functional investigation has just begun. A few studies have thus far indicated biological roles of lncRNAs in self-renewal of spermatogonia (72), regulation of a signaling pathway (73), and progression of meiosis (74) during spermatogenesis. We originally revealed the involvement of a testis-specific lncRNA, *lncRNA-Tcam1*, in the regulation of immune-related genes in mouse spermatocyte-derived cells, and thus, the present study paves the way for investigating novel immune molecular mechanisms underpinning spermatogenesis *via* the endocrine system.

## Ethics Statement

Experimental procedures used in this study were approved by the Institutional Animal Use and Care Committee at Hokkaido University.

## Author Contributions

MK and AK designed the study. MK, KO, SM, and AS performed the experiments. MK, KO, SM, AS, HS, and AK analyzed the data and wrote the paper.

## Conflict of Interest Statement

The authors declare that the research was conducted in the absence of any commercial or financial relationships that could be construed as a potential conflict of interest.

## Supplementary Material

Figure S1 Expression of target gene candidates downregulated by *lncRNA-Tcam1* in postnatal testes. Total RNAs were isolated from 7- and 14-day-old mouse testes, and quantitative reverse transcription-polymerase chain reaction (qRT-PCR) was performed for the candidate genes that might be downregulated by *lncRNA-Tcam1*. All the data were normalized to the value of *Aip* gene. Gray and light black bars represent the data in 7- and 14-day-old testes, respectively. None of the indicated genes showed inverse correlation to *lncRNA-Tcam1*.

Figure S2 Expression of target gene candidates upregulated by *lncRNA-Tcam1* in postnatal testes. Quantitative reverse transcription-polymerase chain reaction (qRT-PCR) was performed, and the data are shown as in Supplemental Figure S1. None of the indicated genes exhibited positive correlation to *lncRNA-Tcam1*.

Table S1 Candidate genes initially identified as those suppressed by *lncRNA-Tcam1*. RNA-sequencing revealed 29 genes that were differentially expressed between GC-2spd-Full and GC-2spd-Δprom. UCSC IDs which mapped to the same gene symbol were unified.

Table S2 Candidate genes initially identified as those activated by *lncRNA-Tcam1*. RNA-sequencing revealed 26 genes that were differentially expressed between GC-2spd-Full and GC-2spd-Δprom. UCSC IDs which mapped to the same gene symbol were unified.

Table S3 Ct values of the six final candidate genes that were potentially activated by *lncRNA-Tcam1* in testicular germ cells by quantitative reverse transcription-polymerase chain reaction (qRT-PCR). The value of an internal control (*Aip*) is also indicated.

## References

[1] Jan SZ Hamer G Repping S de Rooij DG van Pelt AM Vormer TL. Molecular control of rodent spermatogenesis. Biochim Biophys Acta (2012) 1822(12):1838–50. 10.1016/j.bbadis.2012.02.008 22366765

[2] Griswold MD. Spermatogenesis: the commitment to meiosis. Physiol Rev (2016) 96(1):1–17. 10.1152/physrev.00013.2015 26537427 PMC4698398

[3] Alves MG Rato L Carvalho RA Moreira PI Socorro S Oliveira PF Hormonal control of Sertoli cell metabolism regulates spermatogenesis. Cell Mol Life Sci (2013) 70(5):777–93. 10.1007/s00018-012-1079-1 23011766 PMC11113727

[4] Chimento A Sirianni R Casaburi I Pezzi V. Role of estrogen receptors and g protein-coupled estrogen receptor in regulation of hypothalamus-pituitary-testis axis and spermatogenesis. Front Endocrinol (2014) 5:1. 10.3389/fendo.2014.00001 24474947 PMC3893621

[5] Smith LB Walker WH. The regulation of spermatogenesis by androgens. Semin Cell Dev Biol (2014) 30:2–13. 10.1016/j.semcdb.2014.02.012 24598768 PMC4043871

[6] Shima JE McLean DJ McCarrey JR Griswold MD. The murine testicular transcriptome: characterizing gene expression in the testis during the progression of spermatogenesis. Biol Reprod (2004) 71(1):319–30. 10.1095/biolreprod.103.026880 15028632

[7] O’Bryan MK de Kretser D Mouse models for genes involved in impaired spermatogenesis. Int J Androl (2006) 29(1):76–89. 10.1111/j.1365-2605.2005.00614.x 16466527

[8] Roy A Matzuk MM. Deconstructing mammalian reproduction: using knockouts to define fertility pathways. Reproduction (2006) 131(2):207–19. 10.1530/rep.1.00530 16452715

[9] Brower JV Lim CH Jorgensen M Oh SP Terada N. Adenine nucleotide translocase 4 deficiency leads to early meiotic arrest of murine male germ cells. Reproduction (2009) 138(3):463–70. 10.1530/REP-09-0201 19556438 PMC3727218

[10] Yan W. Male infertility caused by spermiogenic defects: lessons from gene knockouts. Mol Cell Endocrinol (2009) 306(1–2):24–32. 10.1016/j.mce.2009.03.003 19481682 PMC5438260

[11] Gaucher J Boussouar F Montellier E Curtet S Buchou T Bertrand S Bromodomain-dependent stage-specific male genome programming by Brdt. EMBO J (2012) 31(19):3809–20. 10.1038/emboj.2012.233 22922464 PMC3463845

[12] Yoneda R Takahashi T Matsui H Takano N Hasebe Y Ogiwara K Three testis-specific paralogous serine proteases play different roles in murine spermatogenesis and are involved in germ cell survival during meiosis. Biol Reprod (2013) 88(5):118. 10.1095/biolreprod.112.106328 23536369

[13] Wu YY Yang Y Xu YD Yu HL. Targeted disruption of the spermatid-specific gene Spata31 causes male infertility. Mol Reprod Dev (2015) 82(6):432–40. 10.1002/mrd.22491 25930072

[14] Miao H Miao CX Li N Han J. FOXJ2 controls meiosis during spermatogenesis in male mice. Mol Reprod Dev (2016) 83(8):684–91. 10.1002/mrd.22671 27316861

[15] Necsulea A Soumillon M Warnefors M Liechti A Daish T Zeller U The evolution of lncRNA repertoires and expression patterns in tetrapods. Nature (2014) 505(7485):635–40. 10.1038/nature12943 24463510

[16] Washietl S Kellis M Garber M. Evolutionary dynamics and tissue specificity of human long noncoding RNAs in six mammals. Genome Res (2014) 24(4):616–28. 10.1101/gr.165035.113 24429298 PMC3975061

[17] Lanz RB McKenna NJ Onate SA Albrecht U Wong J Tsai SY A steroid receptor coactivator, SRA, functions as an RNA and is present in an SRC-1 complex. Cell (1999) 97(1):17–27. 10.1016/s0092-8674(00)80711-4 10199399

[18] Li W Notani D Ma Q Tanasa B Nunez E Chen AY Functional roles of enhancer RNAs for oestrogen-dependent transcriptional activation. Nature (2013) 498(7455):516–20. 10.1038/nature12210 23728302 PMC3718886

[19] Takayama K Horie-Inoue K Katayama S Suzuki T Tsutsumi S Ikeda K Androgen-responsive long non-coding RNA CTBP1-AS promotes prostate cancer. EMBO J (2013) 32(12):1665–80. 10.1038/emboj.2013.99 23644382 PMC3680743

[20] Yang L Lin C Jin C Yang JC Tanasa B Li W lncRNA-dependent mechanisms of androgen-receptor-regulated gene activation programs. Nature (2013) 500(7464):598–602. 10.1038/nature12451 23945587 PMC4034386

[21] Zhang A Zhao JC Kim J Fong KW Yang YA Chakravarti D LncRNA HOTAIR enhances the androgen-receptor-mediated transcriptional program and drives castration-resistant prostate cancer. Cell Rep (2015) 13(1):209–21. 10.1016/j.celrep.2015.08.069 26411689 PMC4757469

[22] Rinn JL Chang HY. Genome regulation by long noncoding RNAs. Annu Rev Biochem (2012) 81:145–66. 10.1146/annurev-biochem-051410-092902 22663078 PMC3858397

[23] Chen LL. Linking long non-coding RNA localization and function. Trends Biochem Sci (2016) 41(9):761–72. 10.1016/j.tibs.2016.07.003 27499234

[24] Dluzen DF Noren Hooten N Evans MK Extracellular RNA in aging. Wiley Interdiscip Rev RNA (2017) 8(2):1385 10.1002/wrna.1385 PMC5315635 27531497

[25] Kim KM Abdelmohsen K Mustapic M Kapogiannis D Gorospe M RNA in extracellular vesicles. Wiley Interdiscip Rev RNA (2017) 8(4):1413 10.1002/wrna.1413 PMC5474163 28130830

[26] Taylor DH Chu ET Spektor R Soloway PD. Long non-coding RNA regulation of reproduction and development. Mol Reprod Dev (2015) 82(12):932–56. 10.1002/mrd.22581 26517592 PMC4762656

[27] Schmitz SU Grote P Herrmann BG. Mechanisms of long noncoding RNA function in development and disease. Cell Mol Life Sci (2016) 73(13):2491–509. 10.1007/s00018-016-2174-5 27007508 PMC4894931

[28] Dey BM Mueller AC Dutta A. Long non-coding RNAs as emerging regulators of differentiation, development, and disease. Transcription (2014) 5(4):e944014. 10.4161/21541272.2014.944014 25483404 PMC4581346

[29] Flynn RA Chang HY. Long noncoding RNAs in cell-fate programming and reprogramming. Cell Stem Cell (2014) 14(6):752–61. 10.1016/j.stem.2014.05.014 24905165 PMC4120821

[30] Aune TM Spurlock CF. Long non-coding RNAs in innate and adaptive immunity. Virus Res (2016) 212:146–60. 10.1016/j.virusres.2015.07.003 26166759 PMC4706828

[31] Valadkhan S Gunawardane LS. lncRNA-mediated regulation of the interferon response. Virus Res (2016) 212:127–36. 10.1016/j.virusres.2015.09.023 26474526 PMC4744491

[32] Zhao XY Lin JD. Long noncoding RNAs: a new regulatory code in metabolic control. Trends Biochem Sci (2015) 40(10):586–96. 10.1016/j.tibs.2015.08.002 26410599 PMC4584418

[33] Wei S Du M Jiang Z Hausman GJ Zhang L Dodson MV. Long noncoding RNAs in regulating adipogenesis: new RNAs shed lights on obesity. Cell Mol Life Sci (2016) 73(10):2079–87. 10.1007/s00018-016-2169-2 26943803 PMC5737903

[34] Hirose T Mishima Y Tomari Y. Elements and machinery of non-coding RNAs: toward their taxonomy. EMBO Rep (2014) 15(5):489–507. 10.1002/embr.201338390 24731943 PMC4210095

[35] Li W Notani D Rosenfeld MG. Enhancers as non-coding RNA transcription units: recent insights and future perspectives. Nat Rev Genet (2016) 17(4):207–23. 10.1038/nrg.2016.4 26948815

[36] Sakatani S Takahashi R Okuda Y Aizawa A Otsuka A Komatsu A Structure, expression, and conserved physical linkage of mouse testicular cell adhesion molecule-1 (TCAM-1) gene. Genome (2000) 43(6):957–62. 10.1139/g00-071 11195349

[37] Nalam RL Lin YN Matzuk MM. Testicular cell adhesion molecule 1 (TCAM1) is not essential for fertility. Mol Cell Endocrinol (2010) 315(1–2):246–53. 10.1016/j.mce.2009.09.010 19766163 PMC2815265

[38] Kurihara M Shiraishi A Satake H Kimura AP A conserved noncoding sequence can function as a spermatocyte-specific enhancer and a bidirectional promoter for a ubiquitously expressed gene and a testis-specific long noncoding RNA. J Mol Biol (2014) 426(17):3069–93. 10.1016/j.jmb.2014.06.018 25020229

[39] Wang H Wen L Yuan Q Sun M Niu M He Z. Establishment and applications of male germ cell and Sertoli cell lines. Reproduction (2016) 152(2):R31–40. 10.1530/REP-15-0546 27069011

[40] Hofmann MC Hess RA Goldberg E Millán JL. Immortalized germ cells undergo meiosis in vitro. Proc Natl Acad Sci U S A (1994) 91(12):5533–7. 10.1073/pnas.91.12.5533 8202522 PMC44030

[41] Wolkowicz MJ Coonrod SA Reddi PP Millan JL Hofmann MC Herr JC. Refinement of the differentiated phenotype of the spermatogenic cell line GC-2spd(ts). Biol Reprod (1996) 55(4):923–32. 10.1095/biolreprod55.4.923 8879510

[42] Chandrasekaran Y McKee CM Ye Y Richburg JH. Influence of TRP53 status on FAS membrane localization, CFLAR (c-FLIP) ubiquitinylation, and sensitivity of GC-2spd (ts) cells to undergo FAS-mediated apoptosis. Biol Reprod (2006) 74(3):560–8. 10.1095/biolreprod.105.045146 16306425

[43] Onorato TM Brown PW Morris PL. Mono-(2-ethylhexyl) phthalate increases spermatocyte mitochondrial peroxiredoxin 3 and cyclooxygenase 2. J Androl (2008) 29(3):293–303. 10.2164/jandrol.107.003335 18077825

[44] Esakky P Hansen DA Drury AM Moley KH. Modulation of cell cycle progression in the spermatocyte cell line [GC-2spd(ts) Cell-Line] by cigarette smoke condensate (CSC) via arylhydrocarbon receptor-nuclear factor erythroid 2-related factor 2 (Ahr-Nrf2) pathway. Biol Reprod (2014) 90(1):9. 10.1095/biolreprod.113.113225 24258214

[45] Wolfe SA Wilkerson DC Prado S Grimes SR. Regulatory factor X2 (RFX2) binds to the H1t/TE1 promoter element and activates transcription of the testis-specific histone H1t gene. J Cell Biochem (2004) 91(2):375–83. 10.1002/jcb.10748 14743396

[46] Grimes SR Prado S Wolfe SA. Transcriptional activation of the testis-specific histone H1t gene by RFX2 may require both proximal promoter X-box elements. J Cell Biochem (2005) 94(2):317–26. 10.1002/jcb.20320 15526285

[47] Li W Wu ZQ Zhao J Guo SJ Li Z Feng X Transient protection from heat-stress induced apoptotic stimulation by metastasis-associated protein 1 in pachytene spermatocytes. PLoS One (2011) 6(10):e26013. 10.1371/journal.pone.0026013 22022494 PMC3192157

[48] Kurihara M Kimura AP. Characterization of the human TCAM1P pseudogene and its activation by a potential dual promoter-enhancer: comparison with a protein-coding mouse orthologue. FEBS Lett (2015) 589(4):540–7. 10.1016/j.febslet.2015.01.023 25622893

[49] Matsubara S Takahashi T Kimura AP. Epigenetic patterns at the mouse prolyl oligopeptidase gene locus suggest the CpG island in the gene body to be a novel regulator for gene expression. Gene (2010) 465(1–2):17–29. 10.1016/j.gene.2010.06.006 20600704

[50] Murakami K Günesdogan U Zylicz JJ Tang WW Sengupta R Kobayashi T NANOG alone induces germ cells in primed epiblast in vitro by activation of enhancers. Nature (2016) 529(7586):403–7. 10.1038/nature16480 26751055 PMC4724940

[51] Luk AC Chan WY Rennert OM Lee TL. Long noncoding RNAs in spermatogenesis: insights from recent high-throughput transcriptome studies. Reproduction (2014) 147(5):R131–41. 10.1530/REP-13-0594 24713396

[52] Chalmel F Lardenois A Evrard B Rolland AD Sallou O Dumargne MC High-resolution profiling of novel transcribed regions during rat spermatogenesis. Biol Reprod (2014) 91(1):5. 10.1095/biolreprod.114.118166 24740603

[53] Ran M Chen B Li Z Wu M Liu X He C Systematic identification of long noncoding RNAs in immature and mature porcine testes. Biol Reprod (2016) 94(4):77. 10.1095/biolreprod.115.136911 26935596

[54] Carpenter S Aiello D Atianand MK Ricci EP Gandhi P Hall LL A long noncoding RNA mediates both activation and repression of immune response genes. Science (2013) 341(6147):789–92. 10.1126/science.1240925 23907535 PMC4376668

[55] Nishitsuji H Ujino S Yoshio S Sugiyama M Mizokami M Kanto T Long noncoding RNA #32 contributes to antiviral responses by controlling interferon-stimulated gene expression. Proc Natl Acad Sci U S A (2016) 113(37):10388–93. 10.1073/pnas.1525022113 27582466 PMC5027408

[56] Fensterl V Sen GC. Interferon-induced Ifit proteins: their role in viral pathogenesis. J Virol (2015) 89(5):2462–8. 10.1128/JVI.02744-14 25428874 PMC4325746

[57] Elkhateeb E Tag-El-Din-Hassan HT Sasaki N Torigoe D Morimatsu M Agui T. The role of mouse 2’,5’-oligoadenylate synthetase 1 paralogs. Infect Genet Evol (2016) 45:393–401. 10.1016/j.meegid.2016.09.018 27663720

[58] Collazo CM Yap GS Sempowski GD Lusby KC Tessarollo L Vande Woude GF Inactivation of LRG-47 and IRG-47 reveals a family of interferon γ-inducible genes with essential, pathogen-specific roles in resistance to infection. J Exp Med (2001) 194(2):181–7. 10.1084/jem.194.2.181 11457893 PMC2193451

[59] Rodríguez-Contreras D de lT Velasco J Shoemaker CB Laclette JP. The *Taenia solium* glucose transporters TGTP1 and TGTP2 are not immunologically recognized by cysticercotic humans and swine. Parasitol Res (2002) 88(3):280–2. 10.1007/s00436-001-0528-9 11954917

[60] Yamamoto M Okuyama M Ma JS Kimura T Kamiyama N Saiga H A cluster of interferon-γ-inducible p65 GTPases plays a critical role in host defense against *Toxoplasma gondii*. Immunity (2012) 37(2):302–13. 10.1016/j.immuni.2012.06.009 22795875

[61] Terpsidis KI Papazahariadou MG Taitzoglou IA Papaioannou NG Georgiadis MP Theodoridis IT. *Toxoplasma gondii*: reproductive parameters in experimentally infected male rats. Exp Parasitol (2009) 121(3):238–41. 10.1016/j.exppara.2008.11.006 19063884

[62] Wang Y Lian Q Yang B Yan S Zhou H He L TRIM30α is a negative-feedback regulator of the intracellular DNA and DNA virus-triggered response by targeting STING. PLoS Pathog (2015) 11(6):e1005012. 10.1371/journal.ppat.1005012 26114947 PMC4482643

[63] Choi UY Hur JY Lee MS Zhang Q Choi WY Kim LK Tripartite motif-containing protein 30 modulates TCR-activated proliferation and effector functions in CD4+ T cells. PLoS One (2014) 9(4):e95805. 10.1371/journal.pone.0095805 24756037 PMC3995923

[64] Graser A Ekici AB Sopel N Melichar VO Zimmermann T Papadopoulos NG Rhinovirus inhibits IL-17A and the downstream immune responses in allergic asthma. Mucosal Immunol (2016) 9(5):1183–92. 10.1038/mi.2015.130 26732679 PMC7099698

[65] Hsu YL Shi SF Wu WL Ho LJ Lai JH. Protective roles of interferon-induced protein with tetratricopeptide repeats 3 (IFIT3) in dengue virus infection of human lung epithelial cells. PLoS One (2013) 8(11):e79518. 10.1371/journal.pone.0079518 24223959 PMC3817122

[66] Pontiroli F Dussurget O Zanoni I Urbano M Beretta O Granucci F The timing of IFNβ production affects early innate responses to *Listeria monocytogenes* and determines the overall outcome of lethal infection. PLoS One (2012) 7(8):e43455 10.1371/journal.pone.0043455 22912878 PMC3422257

[67] Chen Q Deng T Han D. Testicular immunoregulation and spermatogenesis. Semin Cell Dev Biol (2016) 59:157–65. 10.1016/j.semcdb.2016.01.019 26805443

[68] Fijak M Meinhardt A. The testis in immune privilege. Immunol Rev (2006) 213:66–81. 10.1111/j.1600-065X.2006.00438.x 16972897

[69] Wang T Zhang X Chen Q Deng T Zhang Y Li N Toll-like receptor 3-initiated antiviral responses in mouse male germ cells in vitro. Biol Reprod (2012) 86(4):106. 10.1095/biolreprod.111.096719 22262694

[70] Satie AP Mazaud-Guittot S Seif I Mahé D He Z Jouve G Excess type I interferon signaling in the mouse seminiferous tubules leads to germ cell loss and sterility. J Biol Chem (2011) 286(26):23280–95. 10.1074/jbc.M111.229120 21515676 PMC3123094

[71] Yoneda R Satoh Y Yoshida I Kawamura S Kotani T Kimura AP. A genomic region transcribed into a long noncoding RNA interacts with the *Prss42/Tessp-2* promoter in spermatocytes during mouse spermatogenesis, and its flanking sequences can function as enhancers. Mol Reprod Dev (2016) 83:541–57. 10.1002/mrd.22650 27111572

[72] Li L Wang M Wu X Geng L Xue Y Wei X A long non-coding RNA interacts with Gfra1 and maintains survival of mouse spermatogonial stem cells. Cell Death Dis (2016) 7:e2140. 10.1038/cddis.2016.24 26962690 PMC4823932

[73] Arun G Akhade VS Donakonda S Rao MR. mrhl RNA, a long noncoding RNA, negatively regulates Wnt signaling through its protein partner Ddx5/p68 in mouse spermatogonial cells. Mol Cell Biol (2012) 32(15):3140–52. 10.1128/MCB.00006-12 22665494 PMC3434522

[74] Anguera MC Ma W Clift D Namekawa S Kelleher RJ Lee JT. Tsx produces a long noncoding RNA and has general functions in the germline, stem cells, and brain. PLoS Genet (2011) 7(9):e1002248. 10.1371/journal.pgen.1002248 21912526 PMC3164691

