# Supplementary figures and images for: A Testis-Specific Long Non-Coding RNA, lncRNA-Tcam1, Regulates Immune-Related Genes in Mouse Male Germ Cells
Source: Front Endocrinol (Lausanne). 2017 Nov 2;8:299. doi: 10.3389/fendo.2017.00299 (PMC5673629; doi:10.3389/fendo.2017.00299)

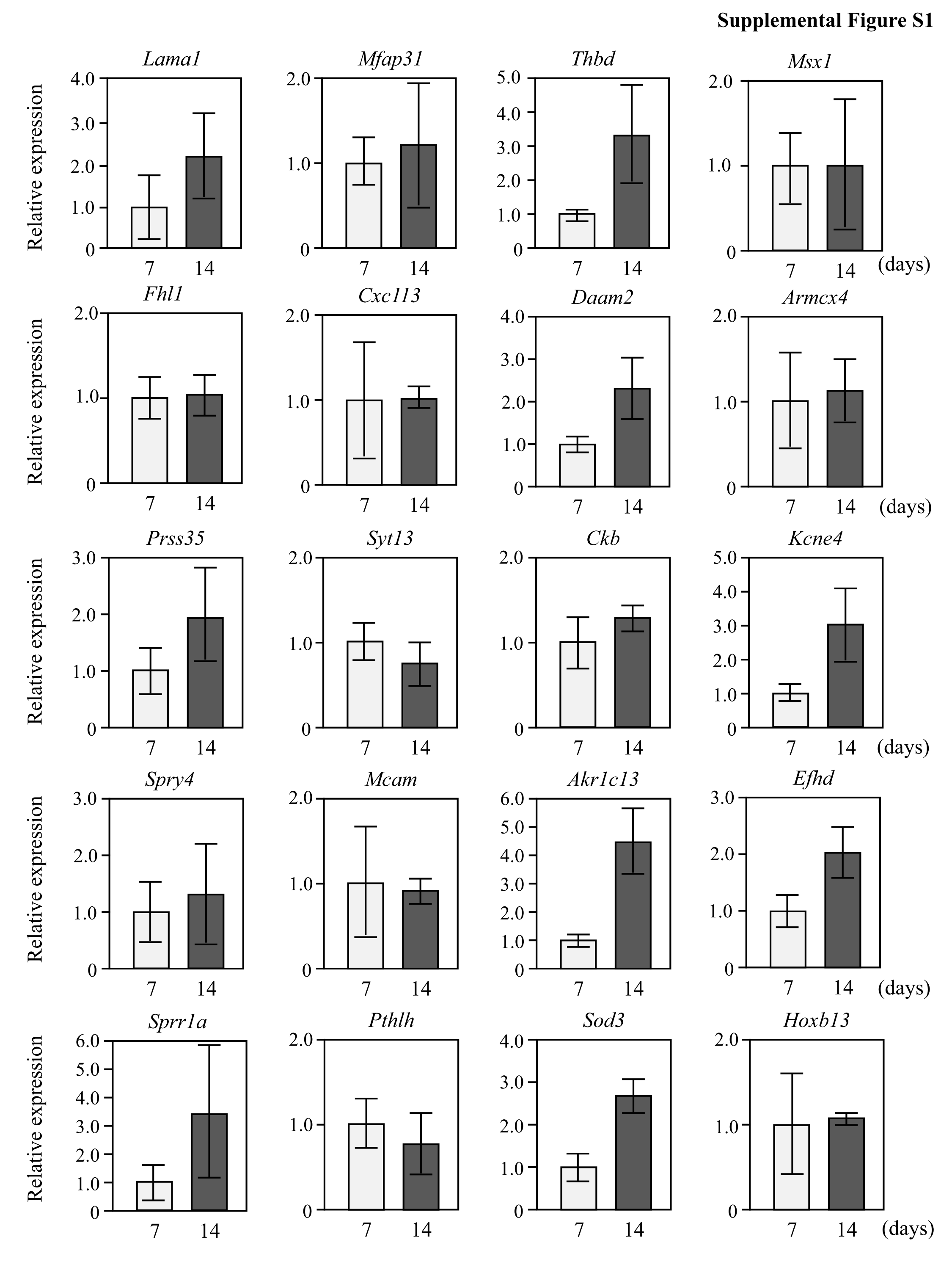

Supplement: Figure S1 — Expression of target gene candidates downregulated by lncRNA-Tcam1 in postnatal testes. Total RNAs were isolated from 7- and 14-day-old mouse testes, and quantitative reverse transcription-polymerase chain reaction (qRT-PCR) was performed for the candidate genes that might be downregulated by lncRNA-Tcam1. All the data were normalized to the value of Aip gene. Gray and light black bars represent the data in 7- and 14-day-old testes, respectively. None of the indicated genes showed inverse correlation to lncRNA-Tcam1. [file Image_1.TIF]

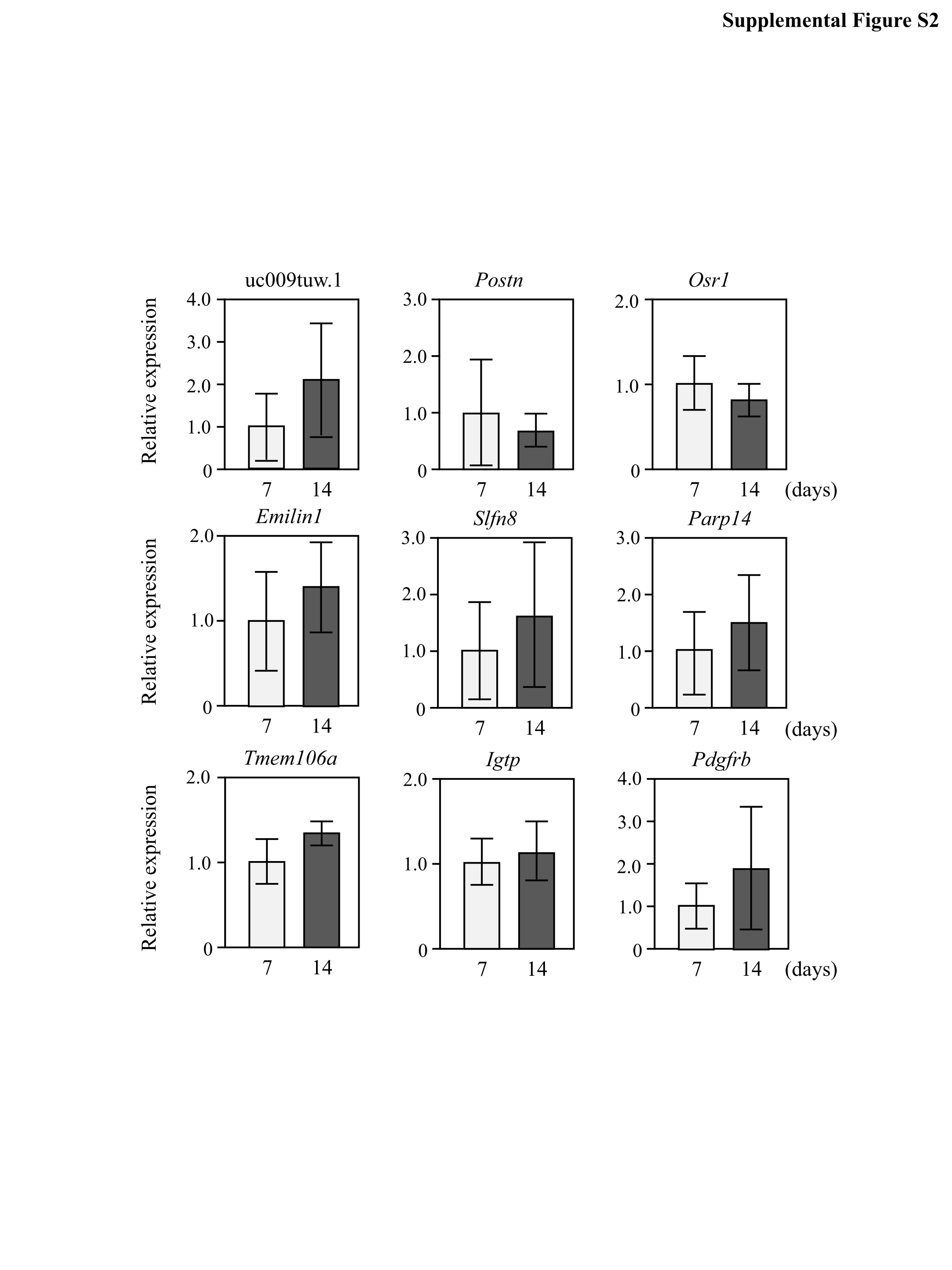

Supplement: Figure S2 — Expression of target gene candidates upregulated by lncRNA-Tcam1 in postnatal testes. Quantitative reverse transcription-polymerase chain reaction (qRT-PCR) was performed, and the data are shown as in Supplemental Figure S1. None of the indicated genes exhibited positive correlation to lncRNA-Tcam1. [file Image_2.TIF]
